# Prevalence of non-communicable diseases and its risk factors among Ijegun-Isheri Osun residents in Lagos State, Nigeria: a community based cross-sectional study

**DOI:** 10.1186/s12889-020-09349-2

**Published:** 2020-08-18

**Authors:** Israel Oluwaseyidayo Idris, Ayodipupo Sikiru Oguntade, Ekow Adom Mensah, Noriko Kitamura

**Affiliations:** 1 Department of Population Health, Faculty of Epidemiology and Population Health, London School of Hygiene and Tropical Medicine, University of London, London, UK; 2 Disease Control and Surveillance Team, Department of Primary Health Programmes, NAIJAHEALTH Initiative, Lagos, Nigeria; 3 Department of Social and Preventive Medicine, V. N Karazin Kharkiv National University, Kharkiv, Ukraine; 4 Health Policy and Governance Unit, Department of State Management and Public Administration, Kharkiv National University of Economics, Kharkiv, Ukraine; 5 Department of Medicine, University College Hospital, Ibadan, Nigeria; 6 Institute of Cardiovascular Science, University College London, London, UK; 7 Department of Family Medicine, Korle-Bu Polyclinic, Accra, Ghana; 8 Institute of Tropical Medicine, Nagasaki University, Nagasaki, Japan; 9 Department of Disease Control, Graduate School of Public Health and Tropical Medicine, Tulane University, New Orleans, USA

**Keywords:** Non-communicable diseases, Hypertension, Dyslipidaemia, Smoking, Physical activity, Risk factors

## Abstract

**Background:**

The rapid epidemiologic transition of diseases has adverse implications for low-and middle-income countries (LMICs) like Nigeria due to their limited healthcare, weaker health systems and the westernization of lifestyle. There is a need to evaluate the enormity or otherwise of non-communicable diseases (NCDs) burden in such low resource settings. We performed this survey to determine the prevalence of NCDs and its risk factors among the Ijegun- Isheri Osun community residents of Lagos, Nigeria.

**Methods:**

A community-based cross-sectional survey was performed on 215 respondents recruited consecutively during a population preventive health campaign. Prevalence of three NCDs (hypertension, diabetes and dyslipidaemia) were calculated. Associations between each of these NCDs and selected risk factors were determined using chi square test. Multivariable logistic regression was used to estimate the risk factors of each of the three NCDs.

**Results:**

The prevalence of hypertension was 35.3% (95% CI 29.0–42.1), diabetes 4.6% (95% CI 2.2–8.4) and dyslipidaemia 47.1% (95% CI 41.1–54.8). Among the NCD risk factors, the prevalence of smoking was 41.3% (95% CI 34.2–48.6), alcohol consumption 72.5% (95% CI 65.5–78.7), and physical activity 52.9 (95% CI 45.5–60.2). The independent significant predictors of hypertension were age ≥ 60 years (aOR 4.56; 95% CI: 1.72–12.09) and dyslipidaemia (aOR 5.01; 95% CI: 2.26–11.13). Age ≥ 60 years (aOR 8.83; 95% CI: 1.88–41.55) was an independent predictor of diabetes. Age ≥ 60 years (aOR 29.32; 95% CI: 4.78–179.84), being employed (aOR 11.12; 95% CI: 3.10–39.92), smoking (aOR 2.34; 95% CI: 1.03–5.33) and physical activity (aOR 0.34; 95% CI: 0.15–0.76) were independent predictors of having dyslipidaemia.

**Conclusions:**

The prevalence of hypertension, diabetes and dyslipidaemia and their associated risk factors are high among the respondents of Ijegun-Isheri Osun community of Lagos state, Nigeria. This highlights the need for further implementation research and policy directions to tackle NCD burden in urban communities in Nigeria. These strategies must be community specific, prioritizing the various risk factors and addressing them accordingly.

## Background

The burden of non-communicable diseases (NCDs) continues to increase globally especially in developing countries, with different risk factors contributing to the surge [1]. This is a result of rapid urbanisation, and westernisation of lifestyle and dietary habits [2–7]. Low-and middle-income countries (LMICs) are likely to suffer a greater burden of these diseases compared to the developed nations because of their limited healthcare financing for NCDs, and their relatively weak and unprepared health systems for these diseases [7–10].

Africa is expected to have the world’s largest increase in NCD deaths over the next decade due to the epidemiologic transition of disease [2]. In most countries in Sub Saharan Africa, NCDs are now responsible for more than three-quarters of all deaths and this will continue to increase if urgent and evidence-based policies to curtail the trend are not successfully implemented [8, 9, 11]. Furthermore, there are clear gaps in the evidence for successful implementation of NCD interventions within the primary health care setting in Sub Saharan African countries. Models and approaches to properly inform the design of interventions that address the needs of communities and individuals are often lacking in most developing countries like Nigeria [12]. For many years, public health policies in Nigeria have focused on the control of infectious diseases, with attendant dearth of necessary data for policy decisions on NCDs [13]. There is a need to pay attention to NCDs in Nigerian communities because these health challenges contribute to economic losses, household poverty and reduction in productivity [14].

Many of the surveys on NCDs in Nigeria have been largely hospital-based. The few available community surveys have shown differences in urban and rural communities in terms of the burden of NCD risk factors. Alikor et al. [15] found that 38% of rural residents in Nigeria had 2 or more NCD risk factors while Odugbemi et al. [16] have reported hypertension prevalence, diabetes, dyslipidaemia and physical inactivity prevalence of 35, 4.6, 47.1 and 92% respectively in traders in a popular Lagos market.

Prevalence rates from direct enumeration of individuals in a representative community provide credible evidence for healthcare planning, allocation of resources and monitoring trajectory of diseases by government agencies. Lagos being the “de facto” industrial capital of Nigeria is a multi-ethnic society undergoing rapid social and lifestyle dynamics of an industrialised urban society. Measuring the prevalence of NCDs in urban communities in Lagos is attractive and provides opportunities for health promotion and policy formulation as it provides a snapshot of the burden of NCDs in such communities.

We thus, set out in this survey to determine the prevalence of NCDs (hypertension, diabetes and dyslipidaemia) and their behavioural risk factors among the Ijegun-Isheri Osun community residents of Lagos, Nigeria.

## Methods

### Study design and setting

This cross-sectional survey was carried out by the NAIJAHEALTH Initiative, a self-funded Non-Governmental Organisation that engages in health promotion in Lagos, Nigeria. The study respondents were residents of the Ijegun- Isheri Osun community, Lagos State, Nigeria who participated in a population preventive health campaign on the 29th and 30th of June, 2018. The sites for the campaign were the Isheri Osun primary healthcare centre, Isheri Osun community market and the Christ apostolic worship centre, Isheri Osun. Residents of this community who were at least 15 years old were recruited consecutively into the study after giving informed consent. Participants who had severe cognitive impairment that mitigated against remembering past events or giving reliable medical history were excluded from the study.

### Sample size

Minimum sample size was calculated using the formula [17]:

$$ N=\frac{{\left({\mathrm{Z}}_{\upalpha /2}\right)}^2\mathrm{pq}}{{\mathrm{d}}^2} $$

Where,

N = the minimum sample size

Z_α/2_ = the standard normal deviate corresponding to a level of significance of 0.05 is 1.96

p = the prevalence rate of hypertension in Abia communities by Ogah *et al* [18] i.e. 31.8%.

q = 1-p

d = the desired precision: 10%

Applying the formula, the minimum sample size is:

$$ {\displaystyle \begin{array}{l}\mathrm{N}=\frac{(1.96)^2\left(0.318\ast 0.682\right)}{(0.1)^2}\\ {}\mathrm{N}=83.3\end{array}} $$

All the 215 respondents who participated in the survey were included in this analysis.

## Data collection

### Data collection instrument

A semi structured interviewer administered questionnaire was developed for this survey (see Additional file 1). The questionnaire was based on the modified WHO Stepwise protocol which consists of three steps (screening questionnaire, physical indices measurement and biochemical measurement) [19, 20]. This is a recommended protocol for epidemiologic studies as it emphasizes collection of good quality data even if the data is small. The questionnaire was divided into subsections of demographic data, medical history, lifestyle risk factors, presenting symptoms and signs, clinical measurements and laboratory test results (see Additional file 1 for further details). The questionnaire was pre-tested before the main study among 10 respondents. The questionnaire was also translated into the Yoruba language which is the local language spoken by most of the populace in the study community. The responses in Yoruba were then translated back into English language before statistical analyses.

### Data collection procedure

The study questionnaire as described was used for data collection. This was administered by trained assistants while medical care and advice was given by clinicians involved in the study.

Blood pressure measurements were obtained with a mercury sphygmomanometer according to standard guidelines [21]. Systolic and diastolic blood pressures were measured at Korotkoff sounds phase I and V, respectively. Two readings were taken at intervals of at least 2 min, and the average of the readings was used to represent the patient’s blood pressure [19]. If there was > 5 mmHg difference between the first and second readings, additional reading was obtained, and then the average of these multiple readings was used [22, 23]. A participant was considered to have hypertension on the basis of self-reported history of hypertension and/or the use of blood pressure-lowering medication and/or documented blood pressure ≥ 140/90 mmHg [18]. Blood pressure status was categorized into normal, pre-hypertension and elevated blood pressure readings using the cut-offs of the European Society of Cardiology [24].

Diagnosis of diabetes was based on self-reported history and/or plasma random glucose reading ≥200 mg/dl with clinical signs of diabetes according to the American Diabetes Association [25]. Smoking status was categorized in to 3 categories. A ‘never smoker’ was someone who had not smoked cigarette in the last 10 years. Occasional or irregular smoker was someone who had smoked at least once in the last 10 years while a regular smoker was defined as someone who smoked at least once a week in the last 10 years.

Alcohol consumption was categorized into three categories. The category ‘Never’ was someone who had never drunk alcohol. Occasional or irregular consumption was ≤monthly consumption and/or 1–2 drinks per day while regular alcohol consumption was defined as drinking > 2 drinks per day. A drink was defined as a bottle or one glass of wine or a shot of spirit.

Regular physical activity was defined as at least 150 min of moderate intensity exercise per week. Irregular physical activity was defined as 30–149 min of moderate intensity physical activity per week while “never” was defined as < 30 min of moderate intensity physical activity per week [26, 27].

Plasma blood glucose and lipids (total cholesterol and LDL) using finger-prick blood sample were measured with point-of-care devices. Normal total plasma cholesterol was defined as < 200 mg/dl while normal LDL was defined as < 130 mg/dl. Dyslipidaemia was defined as plasma cholesterol ≥200 mg/dl and/or LDL cholesterol ≥130 mg/dl.

Those who were diagnosed with the conditions reported were counselled and they were provided with health education. They were then referred to the nearest general hospital for follow up. The NAIJAHEALTH program also has volunteers who followed up these individuals in the community through phone calls and home visitations if required.

## Data analysis

Data were analysed using Stata version 15 (StataCorp LLC, Lakeway Drive, College Station, Texas, USA). Normality of data was determined using Shapiro-Wilk test. Proportions were used to summarize categorical data while continuous variables were summarized as means (standard deviations) as appropriate. Prevalence of each of the NCDs and cardiovascular risk factors were then calculated. The NCDs were modelled as dependent variables while the socio-demographic and lifestyle risk factors were independent variables. The associations between each of the NCDs, and socio-demographic and lifestyle risk factors were determined using chi square. The odds ratios of each of the NCDs adjusted for the various socio-demographic and epidemiologic cardiovascular risk factors were determined using multivariable logistic regression models. Interaction terms were tested for, in all regression models but there was no significant interaction between any of the variables in the regression models. Finally, Poisson regression analyses were performed to determine the effects of certain co-variates on the clustering of NCDs among the participants. Multivariate models were built using significant variables in initial univariate analyses (purposive confirmatory method) and these models were similar to results obtained by stepwise regression models. The results of the purposive confirmatory approach are presented in this paper. A *p* value < 0.05 was considered statistically significant in all analyses.

## Results

The baseline characteristics of the respondents are shown in Table 1 below. Of the study participants, 41.9% were males and 58.1% were females, with a mean age of 38.4 years. About 11(5.1%) had previous diagnosis of diabetes while 40 (18.6%) had a previous diagnosis of hypertension. Sixty-five individuals (34.4%) frequently consumed more than 2 drinks of alcohol per day while only 14 respondents (7.4%) smoked cigarettes at least once a week. One-fifth of the respondents reported regular physical activity or exercise per week. More than half of those tested had elevated plasma cholesterol level. Among the 171 individuals with blood pressure readings, 50.9, 8.2 and 40.9% had normal, prehypertension and hypertension readings respectively.

**Table 1:** Participants’ Baseline Characteristics

Variables		Frequency (%) Mean ± SD
Age (years)		38.4 ± 21.6
Sex	Male	90 (41.9%)
	Female	125 (58.1%)
Marital Status	Single	59 (27.4)
	Married	144 (70.0)
	Divorced	4 (1.9)
	Widowed	5 (2.3)
	Others	3 (1.4)
Occupation	Employed	139 (66.5)
	Unemployed	70 (33.5)
Previous DM history	Yes	11 (5.1)
	No	204 (94.9)
Previous Hypertension history	Yes	40 (18.6)
	No	175 (81.4)
Use of antihypertensives	Yes	11 (5.1)
	No	204 (94.9)
Alcohol consumption	Never	52 (27.5)
	Infrequent	72 (38.1)
	Frequent	65 (34.4)
Smoking	Never	111 (58.7)
	Infrequent	64 (33.9)
	Frequent	14 (7.4)
Recommended Physical activity levels	No	89 (47.1)
	Infrequent	62 (32.8)
	Frequent	38 (20.1)
Blood pressure (171 respondents)	SBP (mmHg)	143 ± 24.4
	DBP (mmHg)	85.7 ± 13.5
Blood pressure categories	Normal (< 120/80 mmHg)	97 (50.9)
	Prehypertension (120–139/80-89 mmHg)	14 (8.2)
	Hypertension (≥140/90 mmHg)	70 (40.9)
RBG (mg/dl)		128.7 ± 49.1
Plasma LDL levels (*n* = 188)	Normal	114 (60.6)
	Elevated	74 (39.4)
Plasma Total cholesterol levels (*n* = 188)	Normal	90 (47.9)
	Elevated	98 (52.1)

Table 2 shows the prevalence of the NCDs and their major risk factors. Hypertension prevalence was 35.3% and similar in both sexes, 4.6% of the subjects had diabetes with no sex difference while 47.9% had dyslipidaemia with a female preponderance (52% in women vs. 42.2% in men).

**Table 2:** Prevalence of NCDs and cardiovascular risk factors in the study respondents

	**Total**	***N*** **= 215**		**Male**	***N*** **= 90**		**Female**	***N*** **= 125**	
**Risk factors**	**N**	**(%)**	**95% CI**	**n**	**(%)**	**95% CI**	**n**	**(%)**	**95% CI**
Hypertension	76	35.3	(29.0–42.1)	32	35.6	(25.7–46.3)	44	35.2	(26.9–44.2)
Diabetes mellitus	10	4.6	(2.2–8.4)	4	4.4	(1.2–11.0)	6	4.8	(1.8–10.1)
Dyslipidemia	103	47.9	(41.1–54.8)	38	42.2	(31.9–53.1)	65	52	(42.9–61.0)
	**Total**	***N*** **= 189**		**Male**	***N*** **= 72**		**Female**	***N*** **= 117**	
**Risk factors**	**N**	**(%)**	**95% CI**	**n**	**(%)**	**95% CI**	**n**	**(%)**	**95% CI**
Smoking	78	41.3	(34.2–48.6)	41	56.9	(44.7–68.6)	37	31.6	(23.3–40.9)
Alcohol consumption	137	72.5	(65.5–78.7)	54	75	(63.4–84.5)	83	70.9	(61.8–79.0)
Physical activity	100	52.9	(45.5–60.2)	47	65.3	(53.1–76.1)	53	45.3	(36.1–54.8)

Less than half of the respondents smoked cigarettes with male preponderance (56.9% in men vs. 31.6% in women). About three-quarters of the respondents consumed alcohol with slight male preponderance while about half of the respondents engaged in exercise with male preponderance (65.3% in men vs. 45.3% in women).

Bivariate analysis using chi-square (see Table 3 below) showed significant association between hypertension and age-group categories. Those who were employed were more likely to have hypertension (*p* < 0.001). There was a strong association between hypertension and diabetes with all those who were diabetic also being hypertensives. Both diabetes and dyslipidaemia also showed increased risk with increasing age-group categories. Hypertension and diabetes were each significantly positively associated with having dyslipidaemia while smoking was also associated with dyslipidaemia (*p* = 0.01). Physical activity was negatively associated with dyslipidaemia.

**Table 3:** Bivariate analysis of the association between risk factors and NCDs

	Hypertension		*p-*value	DM		*p-*value	Dyslipidemia		*p-*value
Variables	Yes (%)	No (%)		Yes (%)	No (%)		Yes (%)	No (%)	
**Age**									
< 40 years	14 (18.4)	92 (66.2)		0 (0.0)	106 (51.7)		18 (17.5)	88 (78.6)	
40-60 years	36 (47.4)	39 (28.0)	< 0.001	3 (30.0)	72 (35.1)	< 0.001	53 (51.5)	22 (19.6)	< 0.001
> 60 years	26 (34.2)	8 (5.8)		7 (70.0)	27 (13.2)		32 (31.1)	2 (1.8)	
**Sex**									
Male	32 (42.1)	58 (41.7)	0.96	4 (40.0)	86 (41.9)	0.90	38 (36.9)	52 (46.4)	0.16
Female	44 (57.9)	81 (58.3)		6 (60.0)	119 (58.1)		65 (63.1)	60 (53.6)	
**Marital status**									
Married	67 (89.3)	77 (56.2)	< 0.001	7 (70.0)	137 (67.8)	0.88	91 (90.1)	53 (47.7)	< 0.001
Unmarried	8 (10.7)	60 (43.8)		3 (30.0)	65 (32.2)		10 (9.9)	58 (52.3)	
**Occupation**									
Employed	63 (84.0)	76 (56.7)	< 0.001	7 (70.0)	132 (66.3)	0.81	91 (89.2)	48 (44.9)	< 0.001
Unemployed	12 (16.0)	58 (43.3)		3 (30.0)	67 (33.7)		11 (10.8)	59 (55.1)	
**Hypertension**									
Yes	–	–	–	10 (0.0)	66 (32.2)	< 0.001	62 (60.2)	14 (12.5)	< 0.001
No	–	–	–	0 (0.0)	139 (67.8)		41 (39.8)	98 (87.5)	
**DM status**									
Yes	10 (13.2)	0 (0.0)	< 0.001	–	–	–	9 (8.7)	1 (0.9)	< 0.01
No	66 (86.8)	139 (100.0)		–	–	–	94 (91.3)	111 (99.1)	
**Smoking**									
Yes	34 (44.7)	44 (38.9)	0.43	3 (30.0)	75 (41.9)	0.46	51 (49.5)	27 (31.4)	0.01
No	42 (55.3)	69 (61.1)		7 (70.0)	104 (58.1)		52 (50.5)	59 (68.6)	
**Alcohol consumption**									
Yes	58 (76.3)	79 (69.9)	0.33	7 (70.0)	130 (72.6)	0.86	77 (74.8)	60 (69.8)	0.44
No	18 (23.7)	34 (30.1)		3 (30.0)	49 (27.4)		26 (25.2)	26 (30.2)	
**Physical activity**									
Yes	36 (47.4)	64 (56.6)	0.21	4 (40.0)	96 (53.6)	0.40	45 (43.7)	55 (63.9)	< 0.01
No	40 (52.6)	49 (43.4)		6 (60.0)	83 (46.4)		58 (56.3)	31 (36.1)	
**Dyslipidemia**									
Yes	62 (81.6)	41 (29.5)	< 0.001	9 (90.0)	94 (45.8)	< 0.01	–	–	–
No	14 (18.4)	98 (70.5)		1 (10.0)	111 (54.2)		–	–	–

When blood pressure and age were modelled as continuous variables, systolic (SBP) and diastolic (DBP) blood pressure showed strong positive linear relationship as shown figure 1 (see Additional file 2). Also, each of SBP and DBP showed strong positive linear relationship with age as shown in figures 2 and 3 respectively (see Additional file 2).

Age, being employed and dyslipidaemia were independent predictors of hypertension as shown in Table 4 below. Respondents aged ≥60 years were about 5 times more likely to have hypertension in adjusted analyses. Respondents who were employed were 4 times more likely to have hypertension in unadjusted analysis and this was attenuated to about 2-fold increased odds of hypertension in adjusted analyses. Dyslipidaemia conferred a 10-fold increased likelihood of hypertension in unadjusted analysis, but this was attenuated by half to 5-fold increased likelihood of hypertension in adjusted analyses.

**Table 4:** Independent risk factors of each of the 3 NCDs by multivariable logistic regression

Variables	Crude OR (95% CI)	*p*-value	Adjusted OR (95% CI)	*p*-value
Hypertension (adjusted for age, occupation and dyslipidemia)				
Age (≥60 years)	8.51 (3.61–20.06)	< 0.05	4.56 (1.72–12.09)	< 0.05
Employed	4.01 (1.98–8.11)	< 0.05	2.29 (0.91–5.77)	0.08
Dyslipidemia	10.58 (5.34–21.00)	< 0.05	5.01(2.26–11.13)	< 0.05
Diabetes (adjusted for age and dyslipidemia)				
Age (≥60 years)	15.38 (3.75–63.12)	< 0.05	8.83 (1.88–41.55)	< 0.01
Dyslipidemia	10.63 (1.32–85.42)	< 0.05	3.89 (0.39–38.65)	0.25
Dyslipidemia (adjusted for age, occupation, smoking, physical activity and hypertension				
Age (≥60 years)	24.79 (5.76–106.67)	< 0.05	29.32 (4.78–179.84)	< 0.05
Employed	10.17 (4.89–21.15)	< 0.05	11.12 (3.10–39.92)	< 0.05
Smoking	2.14 (1.18–3.89)	< 0.05	2.34 (1.03–5.33)	< 0.05
Physical activity	0.44 (0.24–0.79)	< 0.05	0.34 (0.15–0.76)	< 0.05
Hypertension	10.58 (5.34–21.00)	< 0.05	4.31 (1.90–9.77)	< 0.05

Both age and dyslipidaemia were predictors of diabetes in crude analyses, but in adjusted analyses, only age was an independent predictor of diabetes conferring 8.8-fold increased odds of diabetes.

Age, being employed, dyslipidaemia, smoking and hypertension were adverse predictors for having dyslipidaemia while physical activity was protective of having dyslipidaemia in both crude and adjusted analyses. Individuals aged ≥60 years were 30 times more likely to have dyslipidaemia, those who were employed had 11-fold increased odds of having dyslipidaemia, those who smoked were twice at increased likelihood of dyslipidaemia while those with hypertension had 4-fold increased odds of dyslipidaemia. Individuals who engaged in physical activity were 3 times less likely to have dyslipidaemia.

In multivariable Poisson regression, individuals aged ≥60 years had a mean of 2 NCDs while those employed, smokers and the physically inactive each had a mean of 1 NCD as shown in Table 4 (see Additional file 2).

## Discussion

Non-communicable diseases have been projected to be a leading cause of morbidity and mortality in Nigeria by 2030 [28, 29]. The surveillance of NCD risk factors is one of the key strategies advocated to tackle these emerging public health concerns, particularly in low and middle income countries. This study investigated the prevalence of NCDs and their association with behavioural risk factors in the busy surburb of the Ijegun- Isheri Osun in Lagos State, Nigeria. We found that most of the participants were aged 40 years and above, more than half were female, and majority were married.

Tobacco use, the leading cause of morbidity and mortality globally that claims about 6 million lives annually, was also highly prevalent in this study [1, 30]. This finding corroborates reports in India and Bangladesh where prevalence of tobacco use was found to be 34.4 and 43.2% resepectively [31]. It is known that unhealthy lifestyle habits are prevalent in urban cities and industrial hubs in Nigeria [32, 33]. Conversely, low prevalence rates have been reported among the working class in some other parts of Nigeria [29, 34, 35]. These differences could be as a result of civil servants being the focus of previous studies as against this study which participants were general community residents and traders. The sex differences in smoking in this study is in keeping with similar reports in other parts of the country where more males smoked cigarettes than females. This can be attributed to the risk-taking behaviours of men. There was a significant association between increasing age and smoking, which was similar to other existing reports [19]. Similarly previous health reports have concluded that majority of adult smokers initiated the habit of smoking before the age of 18 years, a finding which supports calls for the extension of tobacco control programs to young adults in order to curtail the habit of smoking as people get older [36, 37].

Alcohol consumption and harmful use of alcohol were reported in 32.8 and 34.4% of the respondents respectively. Approximately 2.3 million die each year from the harmful use of alcohol, accounting for about 3.8% of all deaths in the world. More than half of these deaths occur from NCDs including cancers, cardiovascular disease and liver cirrhosis [1]. A prevalence of 26.9% for alcohol consumption has been reported in urban communities in Ibadan [19]. The differences in our findings and that of the earlier studies could be due to differences in study populations, the sampling techniques used, and the prevailing lifestyles present in these different communities. Another reason for inconsistent results with the available literature could be due to differences in assessment methods. Previous studies used questionnaires that were not based on the WHO Stepwise protocol while this study used a pretested questionnaire based on the WHO Stepwise protocol [20].

In our study, incidence of physical activity among the respondents was high (52.9%), which was consistent with previous studies in Ibadan and Abuja, Nigeria which have reported physical activity of 53.6 and 49% respectively among drivers [38, 39]. It is interesting that the commonest occupation among Ijegun-Isheri Osun residents is driving. This is especially in keeping with the higher physical activity observed in males. Also, majority of the female respondents in this study were traders who usually sit in their shops throughout the day as reported also in a study in Tejuosho market in Lagos [16]. Moreover, Lagos is a boisterous city and residents have to engage in demanding jobs and travel long distance daily in search of their daily means of sustenance. However, lower prevalence of physical activity (37.8%) among civil servants in Ibadan have been reported [40, 41]. The differences in the findings could be due to the subjective method of assessment in self-reported questionnaires [42]. Meanwhile, this study used WHO recommendation to classify participants into physical activity categories [43, 44]. One plausible reason for high percentage of physical activity in this study was because most participants were young adults with an average age of 38 years. Also, most of them were employed, even though their occupation were not disclosed but leaving home for work place every day might require walking among most participants. This underscores the importance of workplace interventions that encourage physical activity.

Raised blood pressure, the major risk factor for cardiovascular diseases (CVDs) has become a global concern. This is because CVDs are the leading cause of death globally with an estimated 17.5 million deaths yearly, occurring mostly in low-and-middle income countries [1]. This fact was buttressed in this study where about a third had hypertension, which supports previous findings in other researches in Nigeria [39, 45].This is also in keeping with the landmark meta-analysis by Adeloye et al. [46] who reported prevalence of 30.6% in urban communities in Nigeria. Increasing age has been shown to be a risk factor for raised blood pressure [39, 45]. Participants aged 60 years or above were about nine times more likely to be hypertensive compared to those in age group below 60 years.

As a result of aging, changes occurring within the cardiovascular system like thickening of the arterial wall. Thus, the heart does more work in pushing blood against the thickened arterial wall leading to an increase in arterial blood pressure [40, 47].However, our study did not show any significant gender difference in the occurrence of hypertension although a systematic review on the current prevalence and pattern of hypertension in Nigeria reported higher prevalence of hypertension among males compared to females [40, 41, 48, 49]. Being employed and having dyslipidaemia were other predictors of hypertension in this study. The boisterous and stressful life of Lagos with attendant traffic gridlock may contribute to the increased risk of hypertension seen among the respondents who were employed. Dyslipidaemia contributes to atherosclerosis through endothelial dysfunction, inflammation and insulin resistance.

The low incidence of diabetes in the respondents of the Ijegun-Isheri Osun community at the time of this survey is similar to the finding by Ajayi et al. [38], Oguoma et al. [50] and Sani et al. [51] but lower than the reports by Oluyombo *et al* [52]*.* in Ekiti and Agaba *et al* [53]*.* However, Odugbemi et al. [16] have reported much lower prevalence of diabetes in Tejuosho market in Lagos. Selection bias in our recruitment strategy may explain the difference between our result and the report by Odugbemi et al. [16] It appears that diabetes prevalence is much higher in Nigeria compared to her neighbouring sub-Saharan countries [54].

Dyslipidaemia was prevalent in almost half of the respondents at the time of the survey. In addition, age and the lifestyle risk factors predicted those with dyslipidaemia. Dyslipidaemia usually co-exists with obesity and both are important in the pathway to hypertension and atherosclerotic vascular disease. Ogunbode et al. [55] have coined a mnemonic termed “WASHED” for NCD lifestyle modification and health education in those with obesity in primary care settings. “WASHED” stands for weight control, alcohol reduction, smoking cessation, health promotion, exercise and diet. We have shown in our study the important role of smoking and physical activity as dominant risk factors of dyslipidaemia. We believe that health promotion and education in the community and primary care settings geared towards smoking cessation, increased physical activity and healthy diet would play critical roles in stemming the tide of atherosclerotic vascular diseases in Nigeria. This will require concerted efforts by stakeholders and policy makers if Nigeria is to achieve the 2025 voluntary targets of the Global NCD Action Plan [53].

This study is an important contribution to the surveillance of NCD risk factors in Nigeria. Even though it is not a nationally representative survey, an assessment of respondents in one community in one of the largest cities in Nigeria can give a minuscular view and snapshot of the drivers of NCDs within the larger population until the time when nationally representative surveys would be conducted in Nigeria.

The study is not without limitations. This was a cross-sectional study and it is difficult to prove temporal associations and causality between the NCDs and the epidemiologic NCD risk factors. A longitudinal study in the future would be helpful to investigate these relationships. Also, our sample size is modest and may have biased some of the estimates.

Furthermore, the consecutive recruitment method used in this study is an important limitation of the study and may have resulted in the recruitment of more individuals with background health challenges and subsequent overestimation of the prevalence and effect sizes of the NCDs reported. Moreover, the reported incidence of the NCDs in this survey only provides a glimpse into the NCDs among the residents of Ijegun-Isheri Osun community at the time of the survey. The estimates are specific for this sample of respondents only. The estimates may be different on different days of the weeks or even different weekend days if there is a differential participation of individuals with chronic medical ailments at different time points. However, by allowing everyone in the community to participate, we provided equal chance for participation in the survey and the reported estimates are similar to other reports in Lagos. Despite this, caution should be exercised in generalising the reported estimates to the whole of the Ijegun-Isheri Osun community or other communities in Lagos.

We depended on the verbal reports of the respondents to ascertain their smoking status and alcohol consumption. Some of them may have underreported their previous diagnosis of hypertension or diabetes, use of tobacco and alcohol consumption. We however measured their blood pressure and plasma glucose levels which were also used in making diagnosis of hypertension and diabetes. It is difficult in epidemiologic surveys to exclude misclassifications of lifestyle behaviours in totality. Although, obesity is one of the four metabolic risk factors of NCDs, it was not assessed in this study. However, we have measured blood cholesterol levels which are more important in the pathophysiologic pathway to cardiovascular diseases [56, 57]. Cross tabulation of behavioural risk factors by socio-demographic factors was not computed which makes it difficult to observe the categories of participants that exhibited one habit more than the other. We perhaps underestimated the level of physical inactivity in our study because of the subjective method of assessment in the use of self-reported questionnaires. For future studies, using more objective means of assessing physical activity like pedometers and accelerometers would give more accurate estimates.

## Conclusion

In conclusion, we have shown the prevalence of common NCDs and their risk factors among respondents of the Ijegun-Isheri Osun community in Nigeria’s most busy city at the time of this survey. Many of these risk factors are modifiable and this underscores the importance of health promotion and education in reducing the burden of NCDs in Nigeria. Larger surveys of these nature are needed for policy formulation. We plan to conduct more surveys across Nigeria’s 6 socio-political regions in the future.

## Supplementary information

**Additional file 1.** Data collection instrument. NAIJAHEALTH survey interviewer administered questionnaire.

**Additional file 2.** Supplementary analyses. Supplementary Table and Figures.

## Data Availability

The datasets used and/or analysed during the current study are available from the corresponding author on reasonable request.

## Abbreviations

aOR: Adjusted odds ratio
CVDs: Cardiovascular Diseases
DBP: Diastolic blood pressure
LMIC: Low- and middle-income countries
NCDs: Non communicable diseases
OR: Odds ratio
SBP: Systolic blood pressure
WASHED: Weight control, alcohol reduction, smoking cessation, health promotion, exercise and diet
WHO: World Health Organisation
