# Supplementary material for: Prevalence of non-communicable diseases and its risk factors among Ijegun-Isheri Osun residents in Lagos State, Nigeria: a community based cross-sectional study
Source: BMC Public Health. 2020 Aug 18;20:1258. doi: 10.1186/s12889-020-09349-2 (PMC7437062; doi:10.1186/s12889-020-09349-2)

DATA COLLECTION INSTRUMENT

**,**

**NAIJAHEALTH SURVEY**

**INTERVIEWER ADMINISTERED QUESTIONNAIRE**

Study ID: ________

Date of survey: |__|__|. |__|__|. |__|__|__|__|

**SECTION A: Demographic Data**

Subject’s Initials: ____________________________________

Address: _____________________________________________________________________________­­­­­­­­­­______

Telephone Number 1: __________________ Telephone Number 2:_________________________

City/Place of Residence (in last 5 years) __________________________

Gender: _1_Male _2_Female Domicile: _1_Rural _2_Urban

Date of birth: |__|__|. |__|__|. |__|__|__|__| Age as at last birthday: |__|__| yrs

Marital Status: _1_Single _2_Married _3_Separated/Divorced _4_Widowed _5_ Other; pls specify____________________________

Educational level:  _1_No-formal _2_Primary _3_Secondary _4_Tertiary _5_Others___________

Occupation: _1_Umeployed _2_farming _3_Artisan _4_Trading _5_Office work/Skilled work

_6_Others_______________________

Estimated Monthly Income (Naira)________________________

**SECTION B:** Medical History

History of Hypertension _1_Yes _2_No If YES, Specify duration ____________

Hypertension on treatment _1_Yes _2_No

Diabetes _1_Yes _2_No Kidney disease _1_Yes _2_No

COPD _1_Yes _2_No Ischaemic heart disease _1_Yes _2_No

Obesity _1_Yes _2_No

Other Medical Conditions (Please specify)

1. __________________________ 2. __________________________
2. __________________________ 4. __________________________

Present Medications

Are you on any routine medications? _1_Yes _2_No

If Yes, pls list the medications: ______________________________________________

_______________________________________________________________________

**SECTIONC: Lifestyle Risk Factors**

**I.** **Smoking**

Have you ever smoked cigarette in the past 10 years? _1_Yes _2_No

If Yes, are you a previous smoker or current smoker? Please tick the boxes below as appropriate

Smoking: _1_Previous _2_Current

How often do you smoke cigarette (If previous or current smoker)? Please tick as appropriate the boxes below.

*_1_Only Once _2_Monthly or less _3_Two to four times a week _4_Four or more times a week _5_Daily*

If previous, when did you stop_______ (years)

If previous, Sticks smoked/day ________________ Duration of cigarette smoking (years) _________

If current, Sticks smoked/day __________________ Duration of cigarette smoking (years) _________

NB: Occasional /Irregular smoker (Has smoked at least once in last 10 years); Regular smoker (Smoking at least once in a week)

**II. Alcohol consumption**

Have you ever consumed alcohol in the past 5 years? _1_Yes _2_No

How often do you have a drink containing alcohol?

*_1_Never _2_Monthly or less _3_Two to four times a week _4_Four or more times a week _5_Daily*

How many drinks containing alcohol do you have on a typical day when you are drinking?

(Note: A drink is half a pint of beer or half a glass of wine or a shot of spirit)

*1 or 2 3 or 4 5 or 6 7 to 9 10 or more*

How often during the past year have you found that you were not able to stop drinking once you had started?

*_1_Never _2_ Less than monthly _3_Monthly _4_ Weekly _5_Daily/almost daily*

How often during the past year have you failed to do what was normally expected of you because of drinking?

*_1_ Never _2_ Less than monthly / monthly _3_ Weekly _4_ Daily / almost daily*

Has a relative or friend, doctor or other health worker been concerned about your drinking or suggested you cut down?

*_1_No, _2_ Yes but not in the past year _3_ Yes during the past year*

NB: ‘Never’ was someone who had never drank alcohol. Occasional or irregular consumption was ≤monthly consumption and/or 1-2 drinks per day while regular alcohol consumption was defined as at least weekly alcohol consumption and/or drinking >2 drinks per day

**III. Physical Activity**

Exercise: Do you engage in physical activity of similar or more intensity than brisk walking, cycling or swimming?

_1_Yes _2_ No

If Yes, in a typical week, how many days do you engage in moderate intensity exercise as above? _____________

How much time do you spend/day in moderate intensity exercise (mins)? ____________________

Total time spent in moderate intensity exercise/week (mins) __________________

# D: Symptoms and Clinical Signs

Please as appropriate

1. 2. 3.

4. 5. 6.

7. 8. 9.

**E: Clinical Measurements Examination date: |__|__|. |__|__|. |__|__|__|__|**

# SBP 1 (mmHg) |__|__|__|.|__| SBP 2 (mmHg) |__|__|__|.|__| SBP 3 (mmHg) |__|__|__|.|__|

# DBP 1 (mmHg) |__|__|__|.|__| DBP 2 (mmHg) |__|__|__|.|__| DBP 3 (mmHg) |__|__|__|.|__|

# F: Laboratory Tests Results

# Serum Total Cholesterol |__|__|__| mmol/L Random plasma glucose |__|__|__| mg/dl

# Serum LDL-cholesterol |__|__|__| mmol/L Serum Triglycerides |__|__|__| mmol/L

# THANK YOU FOR PARTICIPATING IN THE NAIJAHEALTH SURVEY

# ADDENDUM

Exercise experts measure activity in metabolic equivalents, or METs. One MET is defined as the energy it takes to sit quietly. For the average adult, this is about one calorie per every 2.2 pounds of body weight per hour; someone who weighs 160 pounds would burn approximately 70 calories an hour while sitting or sleeping.

Moderate-intensity activities are those that get you moving fast enough or strenuously enough to burn off three to six times as much energy per minute as you do when you are sitting quietly, or exercises that clock in at 3 to 6 METs. Vigorous-intensity activities burn more than 6 METs.

One limitation to this way of measuring exercise intensity is that it does not consider the fact that some people have a higher level of fitness than others. Thus, walking at 3 to 4 miles-per-hour is considered to require 4 METs and to be a moderate-intensity activity, regardless of who is doing the activity a young marathon runner or a 90-year-old grandmother. As you might imagine, a brisk walk would likely be an easy activity for the marathon runner, but a very hard activity for the grandmother.

The table below gives examples of light-, moderate-, and vigorous-intensity activity for healthy adults.

#
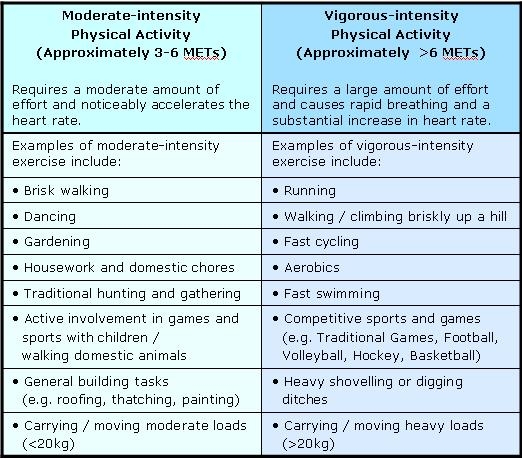

Supplement: Supplementary file 1 — Additional file 1. Data collection instrument. NAIJAHEALTH survey interviewer administered questionnaire. [file 12889_2020_9349_MOESM1_ESM.docx]
