# Supplementary material for: Prevalence of non-communicable diseases and its risk factors among Ijegun-Isheri Osun residents in Lagos State, Nigeria: a community based cross-sectional study
Source: BMC Public Health. 2020 Aug 18;20:1258. doi: 10.1186/s12889-020-09349-2 (PMC7437062; doi:10.1186/s12889-020-09349-2)
Supplement: Supplementary file 2 — Additional file 2. Supplementary analyses. Supplementary Table and Figures. [file 12889_2020_9349_MOESM2_ESM.docx]

**Supplementary analyses**

Table 4: Multivariable Poisson regression of effects of co-variates on clustering of NCDs (R^2^=10.31%)

| Covariates | Mean number of NCDs | Coeff (95%) | IRR (95%CI) | P values |
| --- | --- | --- | --- | --- |
| Age  <60years  ≥60years | 0.75 (0.62-0.87)  1.86 (1.55-2.18) | Ref.  0.91 (0.70-1.13) | Ref.  2.50 (2.01-3.11) | <0.001 |
| Occupation  Unemployed  Employed | 0.55 (0.35-0.75)  1.03 (0.89-1.18) | Ref.  0.63 (0.24-1.02) | Ref.  1.88 (1.27-2.77) | 0.001 |
| Smoking  Never  Regular/irregular | 0.79 (0.63-0.95)  1.02 (0.84-1.21) | Ref.  0.26 (-0.002 – 0.52) | Ref.  1.30 (1.00-1.68) | 0.052 |
| Physical activity  Never  Regular/irregular | 1.08 (0.9-1.26)  0.74 (0.57-0.90) | Ref.  -0.38 (-0.65 to –0.11) | Ref.  0.68 (0.52-0.89) | 0.006 |

Figure 1: Plot of SBP against DBP (r=0.73, p<0.001)

Figure 2: Plot of SBP against age (r=0.54, p<0.001)

Figure 3: Plot of DBP against age (r=0.46, p<0.001)
